# Integrative Analysis of Proteome and Ubiquitylome Reveals Unique Features of Lysosomal and Endocytic Pathways in Gefitinib‐Resistant Non‐Small Cell Lung Cancer Cells

**DOI:** 10.1002/pmic.201700388

**Published:** 2018-07-08

**Authors:** Wang Li, Heyong Wang, Yan Yang, Tian Zhao, Zhixiong Zhang, Ye Tian, Zhaomie Shi, Xiaojun Peng, Fei Li, Yonghong Feng, Lei Zhang, Gening Jiang, Fan Zhang

**Affiliations:** ^1^ Department of Thoracic Surgery Shanghai Key Lab of Tuberculosis Shanghai Pulmonary Hospital School of Life Science and Technology Tongji University Shanghai 200433 P. R. China; ^2^ Jingjie PTM BioLab Co. Ltd. Hangzhou Economic and Technological Development Area Hangzhou 310018 P. R. China; ^3^ Department of Biology New York University New York NY 10003 USA

**Keywords:** proteome, ubiquitylome, gefitinib resistance, non‐small cell lung cancer

## Abstract

Non‐small cell lung cancer (NSCLC) patients carrying EGFR activating mutations treated with gefitinib, a tyrosine kinase inhibitor, will develop drug resistance. Ubiquitylation is one of major posttranslational modifications of proteins affecting the stability or function of proteins. However, the role of protein ubiquitylation in gefitinib resistance is poorly understood. To systematically identify the global change in protein expression and ubiquitylation during gefitinib resistance, a quantitative global proteome and ubiquitylome study in a pair of gefitinib‐resistant and sensitive NSCLC cells is carried out. Altogether, changes in expression of 3773 proteins are quantified, and changes in ubiquitylation of 2893 lysine sites in 1415 proteins are measured in both cells. Interestingly, lysosomal and endocytic pathways, which are involved in autophagy regulation, are enriched with upregulated proteins or ubiquitylated proteins in gefitinib‐resistant cells. In addition, HMGA2 overexpression or ALOX5 knockdown suppresses gefitinib resistance in NSCLC cells by inhibiting autophagy. Overall, these results reveal the previously unknown global ubiquitylome and proteomic features associated with gefitinib resistance, uncover the opposing roles of HMGA2 or ALOX5 in regulating gefitinib resistance and autophagy, and will help to identify new therapeutic targets in overcoming gefitinib resistance.

## 1 Introduction

Gefitinib, a tyrosine kinase inhibitor, is used as a first line treatment against non‐small cell lung cancer (NSCLC) patients with EGFR activating mutations. However, most of these patients will develop gefitinib resistance eventually. Many mechanisms have been reported to regulate gefitinib resistance.^1^, ^2^ However, little is known about how protein ubiquitylation changes during gefitinib resistance.

Protein ubiquitylation is a type of protein posttranslational modification, in which ubiquitin is activated and transferred to substrates via an E1–E2–E3 cascade.^3^ Activated ubiquitin bound via its COOH terminus to E1 is first transferred to E2, which is then transferred to the substrate to form stable protein conjugates in the presence of E3. This process can be repeated many times on the original substrate, leading to the formation of polyubiquitylation chains. Specifically, the C‐terminal glycine in ubiquitin forms the isopeptide bond with the ε‐amino group of lysine residues in substrates. Trypsinolysis of ubiquitylated lysine generates a characteristic “diGLy remnant” due to cleavage of C‐terminal Arg‐Gly‐Gly sequence of ubiquitin, which can be detected and captured by using the antibody recognizing “diGly remnant.”^4^

Ubiquitylation plays important roles in many biological processes including cell cycle, proliferation, and apoptosis. Ubiquitin–proteasome system is responsible for intracellular protein degradation, which serves as the quality control system of the cell.^5^ In addition, ubiquitin modifications have been found to regulate protein interaction, activity, or localization, etc.^6^ For example, polyubiquitylation of PTEN is thought to target PTEN for proteasomal degradation,^7^ while covalent attachment of a single ubiquitin molecule favors its nuclear translocation.^8^ The ubiquitin‐mediated signaling is frequently altered in cancer.^9^ However, little is known about the extent and role of ubiquitylation during gefitinib resistance of NSCLC cells.

**Significance Statement**

In this study, we carried out quantitative proteome and ubiquitylome studies to investigate the global changes in protein expression and ubiquitylation associated with gefitinib resistance in non‐small cell lung cancer (NSCLC) cells. Our results identified thousands of proteins with differential regulation in expression and ubiquitylation associated with gefitinib resistance. In particular, lysosomal and endocytic pathways, which are involved in regulating autophagy, are enriched with proteins with upregulated expression and ubiquitylation. Finally, we found that HMGA2 overexpression or ALOX5 knockdown or inactivation suppressed gefitinib resistance by inhibiting autophagy in these cells. Thus, our study reveals the unique features of gefitinib resistance in both protein expression and ubiquitylation, which will help to identify new therapeutic targets in treating gefitinib resistance in NSCLC.

Lysosomal and endocytic pathways play important roles in the physiology of cells.^10^ They are responsible for communicating between cells and the environment within or from outside, act as platforms for cellular signaling, energy metabolism, and recycling events, and defend against infection or toxic agents, such as drugs. Many regulatory factors are conserved between these two pathways, including those regulating membrane budding and fusion.^10^ For example, Class III PI3K Vps34 plays essential roles in autophagy, endocytosis, and heart and liver function,^11^ Rab7 designates the maturation of endosomes and autophagosomes, directing the trafficking of cargos along microtubules, and participating in the fusion step with lysosomes.^12^ However, the global changes in protein expression and ubiquitylation in these two pathways during gefitinib resistance are not clear.

Here, to identify proteome‐wide changes in protein expression and ubiquitylated proteins associated with gefitinib resistance, we employed antibody‐based capture of endogenous diGly‐containing peptides from cells treated with stable isotope labeling with amino acids in cell culture (SILAC), coupled with liquid chromatography‐tandem mass spectrometry (LC‐MS/MS) analysis, to interrogate the differences in proteome and ubiquitylome between gefitinib‐resistant and sensitive NSCLC cells.

We found that many cellular pathways, especially, lysosomal and endocytic pathways are enriched with upregulated proteins and protein ubiquitylation. In addition, HMGA2 overexpression or ALOX5 knockdown reduces gefitinib resistance by suppressing autophagy. Overall, this study is the first large‐scale mapping of ubiquitylome in gefitinib‐resistant and sensitive NSCLC cells, and will help to identify new therapeutic targets in overcoming gefitinib resistance.

## 2 Experimental Section

### 2.1 Stable Isotope Labeling with Amino Acids in Cell Culture

Gefitinib‐resistant PC9 cells (PC9/GR) were established by the following procedure: nude mice carrying subcutaneous tumor xenografts derived from gefitinib sensitive PC9 cells were intraperitoneally injected with 100 mg kg^−1^ gefitinib for 2 months, the surviving PC9 cells were isolated and expanded in the presence of 3 μm gefitinib in vitro. To show that these PC9/GR cells are free of contaminant cells from the microenvironment of mouse‐resistant cells, we carried out the STR (short tandem repeat) profiling analysis of both PC9 and PC9/GR cells, and showed that they are derived from the common ancestry (PC9 cells). Next, PC9/GR and PC9 cells, were grown to 80% confluence in high glucose (4.5 g L^−1^) Dulbecco's modified Eagle's medium (with glutamine and sodium pyruvate) containing 10% fetal bovine serum and 1% penicillin–streptomycin at 37 °C with 95% air and 5% CO_2_. PC9/GR cells were labeled with “light isotopic lysine” (12C‐lysine), and PC9 cells were labeled with “heavy isotopic lysine” (13C‐lysine) using a SILAC protein quantitation kit (Pierce, Thermo) according to manufacturer's instructions. Briefly, cells were grown in Dulbecco's modified Eagle's medium (the “light” labeled PC9/GR cells were treated with 3 μm gefitinib) supplemented with 10% fetal bovine serum and either the “heavy” form of [U‐13C6]‐ L‐lysine or “light” [U‐12C6]‐ L‐lysine for more than six generations before being harvested, to achieve more than 97% labeling efficiency. After that, the cells were further expanded in SILAC media to desired cell number (≈5 × 10^8^) in fifteen 150 cm^2^ flasks. The cells were then harvested and washed twice with ice‐cold PBS. After snap freezing in liquid nitrogen, cell pellets were stored in −80 °C freezer for future use.

### 2.2 Protein Extraction

The harvested “heavy” and “light” labeled cells were sonicated three times on ice using a high intensity ultrasonic processor (Scientz) in lysis buffer (8 m Urea, 5 mm DTT (Dithiothreitol), 2 mm EDTA, 1.0% cocktail III, 10 mm N‐ethylmaleimide [NEM]). The remaining debris was removed by centrifugation at 20 000 g at 4 °C for 10 min. After concentration measurement, equal amounts of crude proteins in supernatant labeled “heavy” or “light” were mixed and the crude proteins were precipitated by adding TCA with 15% final concentration v/v (soluble fraction). After washing twice with −20 °C acetone, the protein pellets were dissolved in 100 mm NH_4_HCO_3_ (pH 8.0) for trypsin digestion.

### 2.3 Trypsin Digestion

Trypsin (Promega) was added into protein solution with ratio of trypsin to protein at 1:50 w/w for digestion at 37 °C for 16 h. DTT was then added to the final concentration of 5 mm followed by incubation at 50 °C for 30 min. After that, IAA was added to alkylate proteins with the final concentration of 15 mm followed by incubation at the room temperature in dark for 30 min. The alkylation reaction was quenched by 30 mm of cysteine (final concentration) at room temperature for another 30 min. Trypsin was then added again with trypsin to protein ratio at 1:100 w/w for digestion at 37 °C for 4 h to complete the digestion cycle.

### 2.4 High Performance Liquid Chromatography Fractionation

The sample was then fractionated into fractions by high pH reverse‐phase high performance liquid chromatography (HPLC) using Agilent 300 Extend C18 column (5 μm particles, 4.6 mm ID, 250 mm length). Briefly, peptides were first separated with a gradient of 2–60% acetonitrile in 10 mm ammonium bicarbonate pH 10 over 80 min into 80 fractions. Then, the peptides were combined into eight fractions and dried by vacuum centrifuging.

### 2.5 Affinity Enrichment

To enrich Kub (lysine (K) ubiquitylated) peptides, tryptic peptides dissolved in NETN buffer (100 mm NaCl, 1 mm EDTA, 50 mm Tris‐HCl, 0.5% NP‐40, pH 8.0) were incubated with prewashed antibody beads (PTM Biolabs) at 4 °C overnight with gentle shaking. The beads were washed four times with NETN buffer and twice with ddH_2_O. The bound peptides were eluted from the beads with 0.1% TFA. The eluted fractions were combined and vacuum dried. The resulting peptides were cleaned with C18 ZipTips (Millipore) according to the manufacturer's instructions, followed by LC‐MS/MS analysis.

### 2.6 Mass Spectrometer

Thermo Scientific Q Exactive Plus was used here.

### 2.7 Liquid Chromatography‐Tandem Mass Spectrometry Analysis

Peptides were dissolved in 0.1% FA, directly loaded onto a reversed‐phase pre‐column (Acclaim PepMap 100, Thermo Scientific). Peptide separation was performed using a reversed‐phase analytical column (Acclaim PepMap RSLC, Thermo Scientific). The gradient comprised an increase from 6% to 22% of solvent B (0.1% FA in 98% ACN) for 26 min, 22–35% for 8 min, and climbing to 80% in 3 min then holding at 80% for the last 3 min, all at a constant flow rate of 300 nL min^−1^ on an EASY‐nLC 1000 UPLC system, the resulting peptides were analyzed by Q Exactive Plus hybrid quadrupole‐Orbitrap mass spectrometer (ThermoFisher Scientific).

The peptides were subjected to NSI source followed by MS/MS in Q Exactive Plus (Thermo) coupled online to the UPLC. Intact peptides were detected in the Orbitrap at a resolution of 70 000. Peptides were selected for MS/MS using NCE setting as 30; ion fragments were detected in the Orbitrap at a resolution of 17 500. A data dependent procedure that alternated between 1 MS scan followed by 20 MS/MS scans was applied for the top 20 precursor ions above a threshold ion count of 1.0E4 in the MS survey scan with 30.0 s dynamic exclusion. The electrospray voltage applied was 2.0 kV. Automatic gain control (AGC) was used to prevent overfilling of the ion trap; 5E4 ions were accumulated for generation of MS/MS spectra. For MS scans, the *m*/*z* scan range was 350 to 1800.

The mass spectrometry proteomics data have been deposited to the ProteomeXchange Consortium via the PRIDE^13^ partner repository (http://www.ebi.ac.uk/pride/archive/) with the dataset identifier PXD004941.

### 2.8 Protein Quantification

LC‐MS/MS analysis data are further analyzed using the MaxQuant software. Based on the MS/MS spectra, the peptides are identified while the ratios of the according SILAC pairs are used for relative quantification. In each LC‐MS run, we normalize peptide ratios so that the median of their logarithms is zero, which corrects for unequal protein loading, assuming that the majority of proteins show no differential regulation. Protein ratios are calculated as the median of all SILAC peptide ratios, minimizing the effect of outliers. We normalize the protein ratios to correct for unequal protein amounts. Whenever the set of identified peptides in one protein is equal to or completely contained in the set of identified peptides of another protein these two proteins are joined in a protein group. Shared peptides are most parsimoniously associated with the group with the highest number of identified peptides (“razor” peptides) but remain in all groups where they occur. Peptide identification information from the proteomic study is provided (Table S1, Supporting Information).

### 2.9 Database Search

The resulting MS/MS data was processed using MaxQuant with integrated Andromeda search engine (v.1.4.1.2). Tandem mass spectra were searched against Swissprot_human (20 274 sequences) database concatenated with reverse decoy database. Trypsin/P was specified as cleavage enzyme allowing up to four missing cleavages, four modifications per peptide, and five charges. Mass error was set to 10 ppm for precursor ions and 0.02 Da for fragment ions. Carbamidomethylation on Cys was specified as fixed modification and oxidation on Met, ubiquitylation on Lys and acetylation on protein N‐terminal were specified as variable modifications. False discovery rate (FDR) thresholds for protein, peptide and modification site were specified at 1%. Minimum peptide length was set at 7. All the other parameters in MaxQuant were set to default values. The site localization probability was set as >0.75.

### 2.10 Gene Ontology Annotation

Gene Ontology (GO) annotation proteome was derived from the UniProt‐GOA database (http://www.ebi.ac.uk/GOA/). Firstly, converting identified protein ID to UniProt ID and then mapping to GO IDs by protein ID. If some identified proteins were not annotated by UniProt‐GOA database, the InterProScan soft would be used to annotate protein's GO functional based on protein sequence alignment method. Then proteins were classified by Gene Ontology annotation based on three categories: biological process, cellular component, and molecular function.

### 2.11 Domain Annotation

Identified proteins domain functional description were annotated by InterProScan (a sequence analysis application) based on protein sequence alignment method, and the InterPro domain database was used. InterPro (http://www.ebi.ac.uk/interpro/) is a database that integrates diverse information about protein families, domains, and functional sites, and makes it freely available to the public via Web‐based interfaces and services. Central to the database are diagnostic models, known as signatures, against which protein sequences can be searched to determine their potential function. InterPro has utility in the large‐scale analysis of whole genomes and meta‐genomes, as well as in characterizing individual protein sequences.

### 2.12 KEGG Pathway Annotation

First, KEGG online service tool, KAAS, was used to annotate protein's KEGG database description. Then, the annotation results were mapped on the KEGG pathway database using KEGG online service tools KEGG mapper.

### 2.13 Subcellular Localization

Wolf psort, a subcellular localization predication software, was used to predict subcellular localization of proteins. Wolf psort is an updated version of PSORT/PSORT II for the prediction of protein subcellular localization based on their sequences.^14^

### 2.14 Motif Analysis

The motif‐X software was used to analyze the motif of sequences constituted with amino acids in specific positions of modifier‐21‐mers (ten amino acids upstream and downstream of the site) in all protein sequences.^15^ All the database protein sequences were used as background database parameter, other parameters as default.

### 2.15 Enrichment of Gene Ontology Analysis

Proteins were classified by GO annotation into three categories: biological process, cellular compartment, and molecular function. For each category, we used the functional annotation tool of DAVID Bioinformatics Resources 6.7 to identify enriched GO against the background of *Homo sapiens*. A two‐tailed Fisher's exact test was employed to test the enrichment of the protein‐containing IPI entries against all IPI proteins. Correction for multiple hypothesis testing was carried out using standard FDR control methods. The GO with a corrected *p*‐value < 0.05 is considered significant.

### 2.16 Enrichment of Pathway Analysis

Encyclopedia of Genes and Genomes (KEGG) database was used to identify enriched pathways by the functional annotation tool of DAVID against the background of *H. sapiens*. A two‐tailed Fisher's exact test was employed to test the enrichment of the protein‐containing IPI entries against all IPI proteins. Correction for multiple hypothesis testing was carried out using standard FDR control methods. The pathway with a corrected *p*‐value < 0.05 was considered significant. These pathways were classified into hierarchical categories according to the KEGG website.

### 2.17 Enrichment‐Based Clustering

All the protein categories obtained after enrichment were collated along with their *p* values, and then filtered for those categories which were at least enriched in one of the clusters with *p* value < 0.05. This filtered *p* value matrix was transformed by the function *x* = −log10 (*p* value). Finally these *x* values were *z*‐transformed for each category. These *z* scores were then clustered by one‐way hierarchical clustering (Euclidean distance, average linkage clustering) in Genesis. Cluster membership was visualized by a heat map using the “heatmap.2” function from the “gplots” R‐package.

### 2.18 Functional Enrichment‐Based Clustering for Protein Groups

For further hierarchical clustering based on different protein functional classification (such as: GO, Domain, Pathway, Complex). We first collated all the categories obtained after enrichment along with their *p* values, and then filtered for those categories which were at least enriched in one of the clusters with *p* value < 0.05. This filtered *p* value matrix was transformed by the function *x* = −log10 (*p* value). Finally, these *x* values were *z*‐transformed for each functional category. These *z* scores were then clustered by one‐way hierarchical clustering (Euclidean distance, average linkage clustering) in Genesis. Cluster membership was visualized by a heat map using the “heatmap.2” function from the “gplots” R‐package.

### 2.19 Quantiles‐Based Clustering for Protein Groups

The quantified proteins in this study were divided into four quantiles. The average (*x*) and standard deviation (*y*) of the log2 ratio (L/H) of all quantified peptides was calculated. The L/H ratio of each peptide was then transformed to an *z* score based on *z* = (log2ratio – *x*) / *y*, where the ratio is the L/H ratio. The cutoff *z* scores was set according to cumulative density function of normal distribution at three different percentiles — 25, 50 and 75%. Each peptide was then allocated to the quantiles based on the transformed *z* score. In this way, four quantiles were generated: Q1 (0–25%), Q2 (25–50%), Q3 (50–75%), and Q4 (75–100%).

### 2.20 Clustering Method

All the substrate categories obtained after enrichment were collated along with their *p* values, and then filtered for those categories which were at least enriched in one of the clusters with *p* value < 0.01. This filtered *p* value matrix was transformed by the function *x* = −log10 (*p* value). Finally, these *x* values were *z*‐transformed for each category. These *z* scores were then clustered by one‐way hierarchical clustering (Euclidean distance, average linkage clustering) in Genesis. Cluster membership was visualized by a heat map using the “heatmap.2” function from the “gplots” R‐package.

### 2.21 RNA Isolation and Real‐Time RT‐PCR (Reverse Transcription‐Polymerase Chain Reaction)

Total RNA extraction from cells and real‐time RT‐PCR were performed as previously described.^16^ The PCR primers used were as follows: HMGA2 forward, 5ʹ‐ GCCACCCACTACTCTGTCCT‐3ʹ; HMGA2 reverse, 5ʹ‐TTGAGATTGAAAGTGCCTTGG‐3ʹ; ALOX5 forward, 5ʹ‐GATTGTCCCCATTGCCATCC‐3ʹ; ALOX5 reverse, 5ʹ‐AGAAGGTGGGTGATGGTCTG‐3ʹ; GAPDH forward, 5ʹ‐GAGTCAACGGATTTGGTCGT‐3ʹ; and GAPDH reverse, 5ʹ‐ TTGATTTTGGAGGGATCTCG‐3ʹ.

### 2.22 Western Blotting

Western blotting was performed as previously described.^16^ In some experiments, cells were treated with 20 μg mL^−1^ cycloheximide (CHX) (Sigma, #C7698) for 0, 2, 4, and 8 h before harvesting. The following primary antibodies were used: HMGA2 (CST, #8179S), ALDH1A1 (CST, # 12035S), CHK2 (CST, # 3440S), FAS (CST, # 8023S), TGFBR1 (CST, # 3712S), Flag (Sigma‐Aldrich, #F3165), LC3B (CST, #3868), NUCB2 (Proteintech, #26712‐1‐AP), GAPDH (Sigma‐Aldrich, #G9545). GAPDH was used as a loading control. The following secondary antibodies were used: goat anti‐rabbit IgG (CST, #7074S) and goat polyclonal anti‐mouse IgG (Abcam, #ab136815).

### 2.23 Colony Formation Assay

Colony formation assays were performed as previously described.^16^ The assays were performed in triplicate.

### 2.24 Establishment of HMGA2 or ALOX5 Overexpression or Knockdown Cell Lines

PC9/GR cells were infected with the lentiviral supernatant containing constructs overexpressing the control plasmid, HMGA2 or ALOX5 cDNA, or constructs expressing the control shRNA, shRNAs targeting HMGA2 or ALOX5. Puromycin was added to the cells for killing uninfected cells. Multiple single colonies were selected and expanded. The detailed procedure was described as previously described.^17^

### 2.25 Small Molecule Inhibitor

Zileuton (# HY‐14164) was purchased from MedChemExpress Inc. (Monmouth Junction, NJ, USA).

### 2.26 Statistical Analysis

For comparisons between two groups, Student's *t*‐test was used. For comparisons among multiple groups, one‐way ANOVA was used. For all analyses, a *p* value of < 0.05 was considered statistically significant. *p < 0.05, **p < 0.01, ***p < 0.001.

## 3 Results

### 3.1 Characterization of Ubiquitylated Sites in Gefitinib‐Resistant Cells

**Figure 1:**
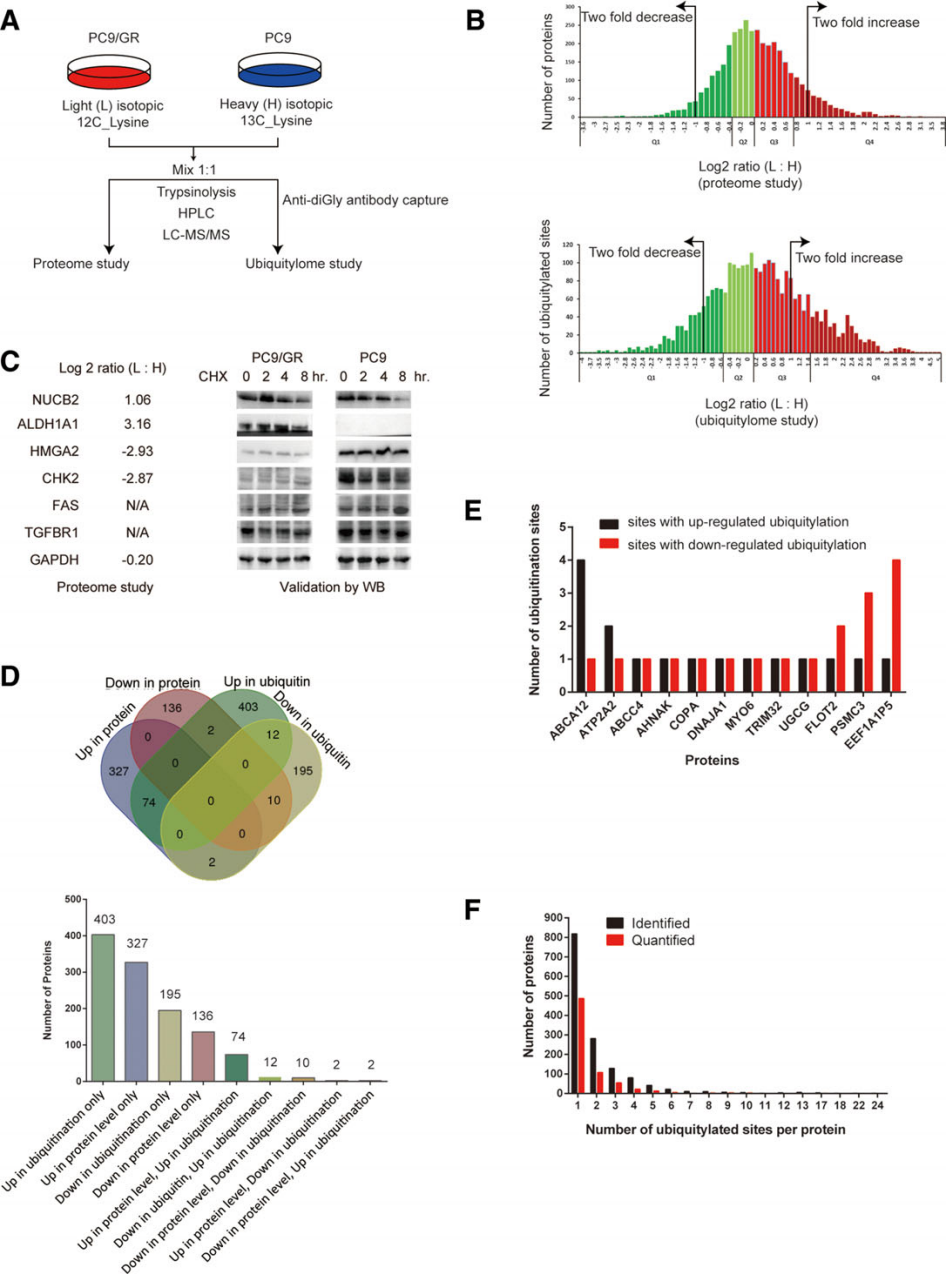
Characterization of protein ubiquitylome in gefitinib‐resistant NSCLC cells. A) Proteome and ubiquitylome project workflow. PC9/GR cells were labeled with light (L) isotopic 12C lysine, and PC9 cells were labeled with heavy (H) isotopic 13C lysine. B) Quantiles‐based clustering of quantified proteins in the proteome study (upper panel) or quantified ubiquitylated lysine sites in the ubiquitylome study (lower panel), in PC9/GR versus PC9 cells. The quantified proteins or ubiquitylated lysine sites were divided into four quantiles according to the log2 ratio (L: H): Q1 (0–25%; dark green), Q2 (25–50%; light green), Q3 (50–75%; light red), and Q4 (75–100%; dark red). C) Comparison of protein expression in PC9/GR versus PC9 cells using two different methods. Log2 ratio (L:H) of indicated proteins from the proteome study (left panel). N/A stands for the data not available in the proteome study. Western blotting (WB) validation of differentially expressed proteins in PC9/GR and PC9 cells (right panel). GAPDH serves as a loading control in WB. D) Venn diagram showing the overlapping groups of proteins among up‐ or downregulated proteins and proteins with up‐ or downregulated ubiquitylated lysine sites in PC9/GR cells versus PC9 cells. The bar graph shows the number of proteins in each overlapping group of proteins. E) Bar graph showing 12 proteins containing both up‐ and downregulated ubiquitylation sites within the same protein. F) Distribution of the number of ubiquitylated lysine sites per protein in PC9/GR versus PC9 cells. The black or red bar indicates proteins either identified or quantified in the ubiquitylome study.

To systematically identify the global change in the ubiquitylated targets of gefitinib‐resistant cells (PC9/GR) and its gefitinib sensitive parental cells (PC9), we carried out both proteome and ubiquitylome studies at the same time (Figure 1A). In this study, we used these two conditions: PC9 cells cultured in the medium without gefitinib, and PC9/GR cells cultured in the medium with gefitinib. We did not choose two other conditions: PC9 cells cultured in the presence of gefitinib, because most of cells would die after several passages, resulting in the lack of starting materials for the subsequent analysis. In addition, we did not choose PC9/GR cells cultured without gefitinib, because we found that PC9/GR cells cultured without gefitinib gradually lost its resistance and proliferated faster over time, which means they become more similar to PC9 cells. This observation was also confirmed by previous studies,^2^ in which they found that there was a chromatin‐mediated reversible gefitinib‐tolerant state in cancer cell subpopulations. Therefore, we believe the two conditions used in this study can provide the most informative information specifically related to gefitinib resistance.

Our ubiquitylome study is essentially a quantitative diGly proteomics. It combines antibody‐based capture of “diGly remnant”‐containing peptides (marking ubiquitylated proteins after trypsinolysis) and SILAC. PC9/GR cells were labeled with light (L) isotopic 12C‐lysine, while PC9 cells were labeled with heavy (H) isotopic 13C‐lysine. These were followed by HPLC and LC‐MS/MS analysis. The normalized ratio of the peptide intensity from these two types of cells (ratio (L:H)) was used to determine the relative peptide abundance in PC9/GR versus PC9 cells. QC validation of the MS data was carried out to determine whether the experimental condition in each study was optimal (Figure S1, Supporting Information).

For global proteome analysis, 4646 proteins were identified and 3773 proteins were quantified in both PC9/GR and PC9 cells. Among them, 404 proteins were upregulated, and 148 were downregulated in PC9/GR cells, when using the normalized log2 ratio (L:H) either ≥1 or ≤−1 (equivalent to twofold change in both directions) as the threshold (Table S1, Supporting Information). Quantiles‐based analysis divides all protein into four groups (Q1, Q2, Q3, and Q4) when ranked by log2 ratio (L:H), and shows that 15.8% of proteins with log2 ratio (L:H) either ≥1 or ≤−1 (Figure 1B, upper panel). Western blotting assays showed that, while NUCB2 was degraded more slowly upon CHX treatment in PC9/GR cells than in PC9 cells, ALDH1A1 was upregulated, HMGA2 and CHK2 were downregulated in PC9/GR cells, and FAS and TGFBR1 had no difference in protein expression between PC9/GR and PC9 cells (Figure 1C). These results are consistent with the MS data in the proteome study (Table S2, Supporting Information).

For ubiquitylome analysis, 2941 ubiquitylated lysine sites in 1432 proteins were identified, and 2893 lysine sites in 1415 proteins were quantified in both PC9/GR and PC9 cells. Among them, 799 ubiquitylated lysine sites were quantified as upregulated targets and 339 ubiquitylated sites were quantified as downregulated targets, when using the normalized log2 ratio (L:H) either ≥1 or ≤−1 as the threshold (Table S2, Supporting Information). A typical secondary MS result was presented here using NBR1 protein as an example (Figure S2, Supporting Information). Quantile‐based analysis divides all protein into four groups (Q1, Q2, Q3, and Q4) when ranked by log2 ratio (L:H), and shows 41.1% of lysine sites with log2 ratio (L:H) either ≥1 or ≤−1 (Figure 1B, lower panel).

Venn diagram analysis reveals that, among these differentially regulated proteins in PC9/GR cells, 74 proteins have both upregulated protein level and ubiquitylation level, and 10 proteins have both downregulated protein level and ubiquitylation level, only two proteins (ALOX5 and NUCB2) have upregulated protein level, but downregulated ubiquitylation level, and two proteins (EPHA2 and CNNM3) have downregulated protein level, but upregulated ubiquitylation level in PC9/GR cells. In addition, 12 proteins have both up‐ and downregulated ubiquitylated sites in PC9/GR cells (Figure 1D). For example, ABCA12 protein has two lysine sites with upregulated ubiquitylation, but one lysine site with downregulated ubiquitylation (Figure 1E). We also found that most of the proteins have only one ubiquitylated site, while few proteins have more than one ubiquitylated site (Figure 1F). Overall, our study reveals the unique characters of the ubiquitylome in PC9/GR cells, compared to PC9 cells.

### 3.2 Construction of the Ubiquitylated Lysine Motifs

**Figure 2:**
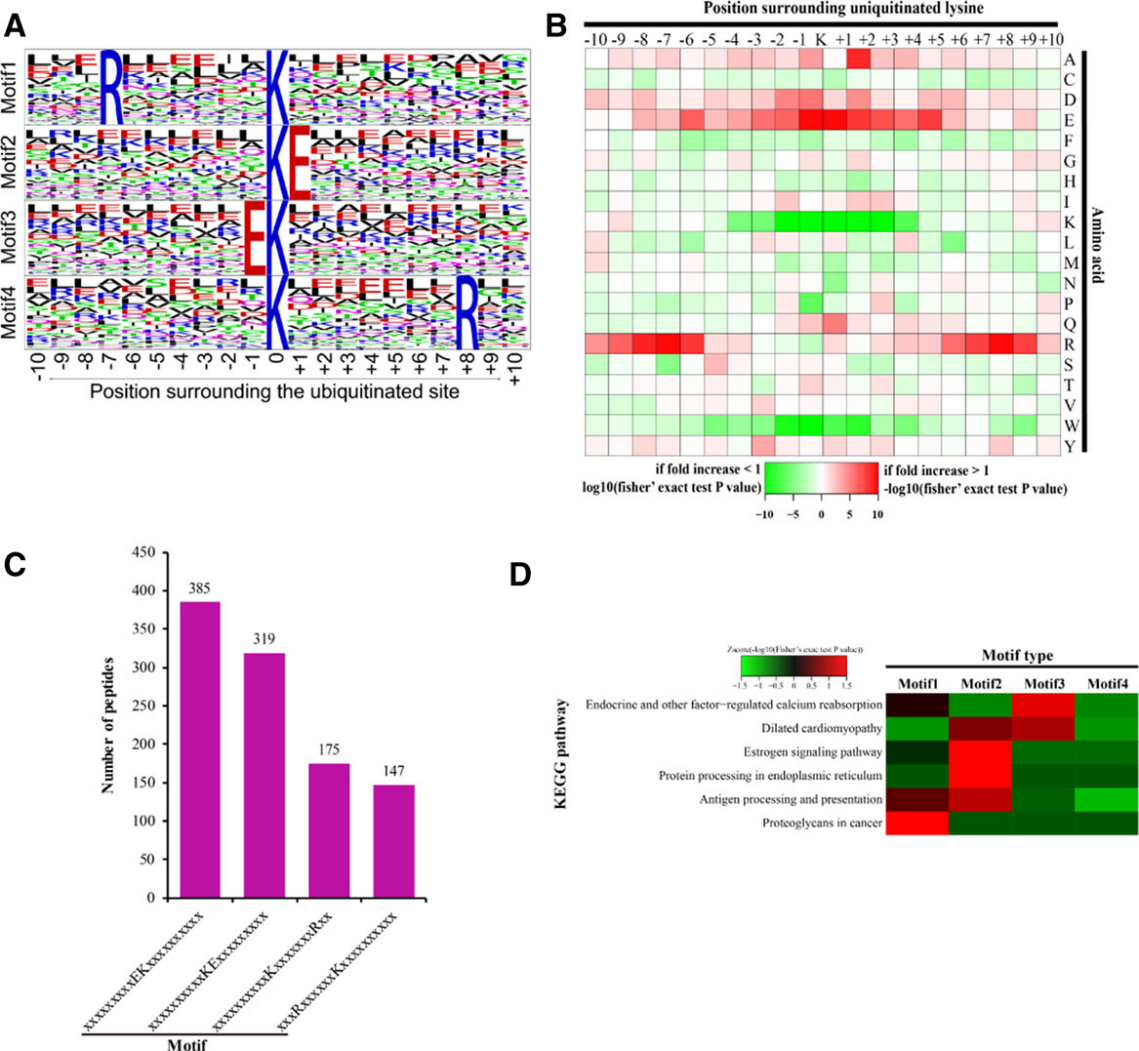
Analysis of the characters of the ubiquitylated peptides in PC9/GR and PC9 cells. A) Ubiquitylation motifs were constructed with the Motif‐X software. The central K (at position 0) indicates the ubiquitylated lysine. All the surrounding amino acid residues are indicated with the letters in different heights which is consistent with their frequency in respective position. B) Heat map showing the frequency of the amino acid residues around the ubiquitylated lysine site based on analysis of all the ubiquitylated peptides in each motif. C) Number of the ubiquitylated peptides in each motif. D) Heat map showing the enriched motifs in the representative KEGG pathways. *z* score = −log10 (Fisher's exact test *p* value). Green represents the negative *z* score, and red represents the positive *z* score.

The motif‐X program was used to analyze the sequence motif of ubiquitylated peptides in PC9/GR and PC9 cells. Of all the ubiquitylated peptides identified (Table S3, Supporting Information), 1026 peptides were found to be enriched in four conserved motifs, within 20 amino acid residues surrounding the ubiquitylated lysine (from the −10 to the +10 positions). These motifs (motif 1–4) are illustrated as RXXXXXXKub, KubE, EKub, and KubXXXXXXXR, respectively (Kub is the ubiquitylated lysine, and X represents a random amino acid residue; Figure 2A).

The analysis of these motifs suggested that two kinds of amino acid residues are enriched in the motifs. These amino acid residues included a positively charged residue (arginine [R]) and a negatively charged residue (glutamate [E]). These motifs can be sorted into two categories: the position +6 or −7 is R, and the position +1 or −1 is E. Notably, the positively charged residue (K, H, or R) is rarely found near the ubiquitylated lysine. These results suggest that ubiquitylation system prefers to modify the lysine residue surrounded with negatively changed residues from position −5 to +5 and without the positively charged residues (Figure 2B).

All the motifs differed in their abundances in the ubiquitylated peptides, and motif 3 has the most extensive distribution, while motif 1 has the least distribution (Figure 2C). Furthermore, enrichment analysis indicated that these four motifs exhibit the different distributions over KEGG pathways. Motif 1 was significantly overpresented in the pathway of proteoglycan in cancer, motif 3 was enriched in the pathway of endocrine and other factor‐regulated calcium reabsorption. However, motif 2 was enriched in at least two pathways, including estrogen signaling and protein processing in endoplasmic reticulum (Figure 2D). Overall, our study reveals the unique features of motifs in ubiquitylated lysine peptides in gefitinib‐resistant cells.

### 3.3 Functional Annotation, Pathway Enrichment, and Clustering Analysis of Protein Ubiquitylation in Gefitinib‐Resistant Cells

**Figure 3:**
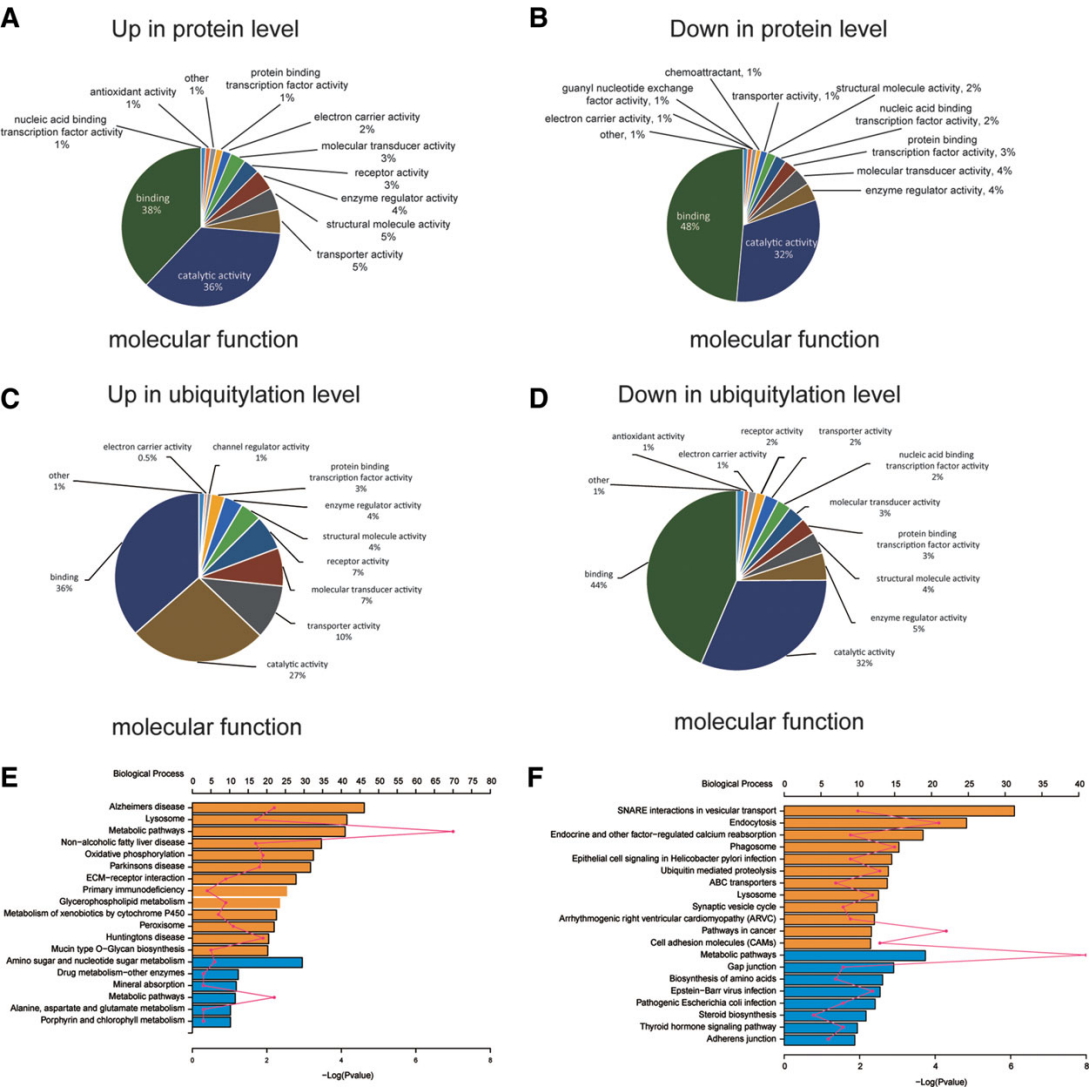
Classification of molecular functions and pathway enrichment analysis for differentially expressed proteins or proteins with lysine sites undergoing differential regulation in ubiquitylation in PC9/GR versus PC9 cells. Molecular functions for (A) upregulated or (B) downregulated proteins in PC9/GR versus PC9 cells. Molecular functions for proteins containing lysine sites undergoing (C) upregulated or (D) downregulated ubiquitylation in PC9/GR versus PC9 cells. Pathway enrichment analysis for (E) differentially expressed proteins or (F) proteins with differentially regulated ubiquitylation in PC9/GR versus PC9 cells. The top *x* axis represents the number of proteins enriched in the pathway, the bottom *x* axis represents – log (*p* value), and the *y* axis represents the name of KEGG pathway.

To understand the functions or pathways of proteins differentially expressed or regulated by ubiquitylation associated with gefitinib resistance, all these proteins were annotated using Gene Ontology (GO). According to molecular function, proteins with increased expression in PC9/GR cells are enriched in catalytic, transporter, structural molecular, receptor, and electron carrier activities, while proteins with downregulated expression are enriched in binding activity in general (Figure 3A,B). By comparison, proteins containing increased ubiquitylated lysine sites in PC9/GR cells are enriched in transporter, molecular transducer, and receptor activities, while proteins with decreased ubiquitylated lysine sites are enriched in binding, catalytic, and enzyme regulatory activities (Figure 3C,D). Among the assigned catalogues, two catalogues (i.e., binding and catalytic activities) contain proteins occupying more than 60% of total proteins undergoing differential regulation in ubiquitylation in PC9/GR cells (Figure 3C,D).

Based on subcellular location, proteins with increased expression in PC9/GR cells are enriched in nuclear, mitochondria, and extracellular compartment, while proteins with downregulated expression in PC9/GR cells are enriched in plasma membrane, cytosol, and nuclear compartments (Figure S3a,b, Supporting Information). In addition, proteins with increased ubiquitylated lysine sites in PC9/GR cells are enriched in peroxisome, nuclear, and mitochondria, but proteins with decreased ubiquitylated lysine sites are enriched in plasma membrane, cytosol, and cytoskeleton (Figure S3c,d, Supporting Information).

In addition, all differentially expressed proteins were subjected to the enrichment analysis with KEGG pathways, which are a collection of pathway maps representing updated knowledge on the molecular interaction and reaction networks.^18^ The analysis showed that 13 pathways were significantly enriched in upregulated proteins, including lysosome, oxidative phosphorylation, ECM (Extracellular Matrix)–receptor interaction, glycerophospholipid metabolism, and metabolism of xenobiotic by cytochrome P450 in PC9/GR cells, while six pathways were enriched in downregulated proteins, including amino sugar and nucleotide sugar metabolism, metabolic pathways in PC9/GR cells (Figure 3E and Table S4, Supporting Information).

Proteins containing differential regulation in ubiquitylation were also subjected to the enrichment analysis with KEGG pathways. The analysis showed that 12 pathways were significantly enriched in proteins with increased ubiquitylation, including SNARE interaction in vesicular transport, endocytosis, phagosome, ABC transporters, lysosome in PC9/GR cells, while 8 pathways were significantly enriched in proteins with decreased ubiquitylation, including metabolic pathways, gap junction, and biosynthesis of amino acids in PC9/GR cells (Figure 3f and Table S4, Supporting Information).

Protein functional domain clustering for previously described four protein groups (Q1, Q2, Q3, and Q4) in the ubiquitylome study was carried out (Figure S4, left panel, Supporting Information). Proteins in the Q1 group, which contain lysine sites with decreased ubiquitylation, are enriched in the following protein domains: Zinc finger C6HC‐type, glutathione S‐transferase, the conserved sequence in the N‐terminal section of class I aminoacyl‐tRNA synthetases, etc. However, proteins in the Q4 group, which contain lysine sites with increased ubiquitylation, are enriched in the following protein domains: transmembrane receptor, E3 ubiquitin–protein ligase SMURF1 type, EGF‐like calcium binding domain, etc. (Figure S4, left panel, Supporting Information).

KEGG pathway clustering for four protein groups (Q1, Q2, Q3, and Q4) in the ubiquitylome study was also carried out (Figure S4, right panel, Supporting Information). Proteins in the Q1 group are enriched in the following KEGG pathways: cell cycle, metabolic pathway, DNA replication, etc. However, proteins in the Q4 group are enriched in the following pathways: SNARE interaction in vesicle transport, endocytosis, lysosome, ABC transporters, etc., which is consistent with previous KEGG enrichment analysis using all proteins with upregulated ubiquitylation in PC9/GR cells (Figure 3F).

These findings reveal that gefitinib resistance is associated with many distinctive cellular pathways with significant up‐ and downregulation of protein expression or ubiquitylation.

### 3.4 Protein Expression and Ubiquitylation are Upregulated in Lysosomal and Endocytic Pathways

**Figure 4:**
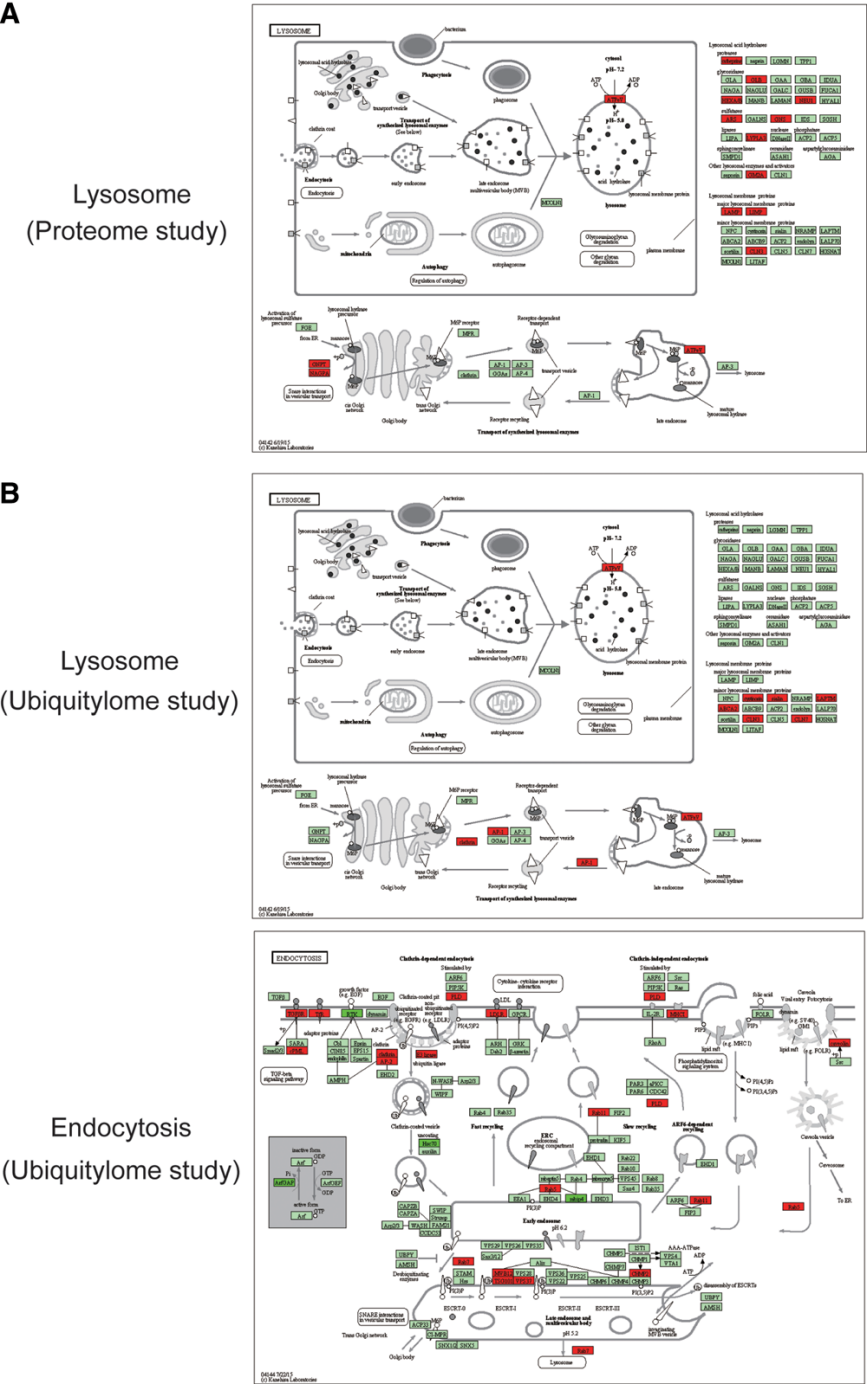
Lysosomal and endocytic pathways are enriched with upregulated proteins or protein ubiquitylation in PC9/GR cells. A) The lysosomal pathway was significantly overpresented with upregulated proteins. B) Lysosomal and endocytosis pathways were significantly overpresented with upregulated protein ubiquitylation in PC9/GR cells. Proteins with upregulated (red), downregulated (dark green), or similar (light green) expression or ubiquitylation (red) in PC9/GR cells are shown.

The lysosomal pathway is enriched with upregulated proteins in gefitinib‐resistant cells. For example, lysosomal membrane proteins, including LAMP1/2 and LIMP, are upregulated. In addition, lysosomal acid hydrolases, such as, CTSA, CTSD, CTSH, GLB1, NEU1, HEXA/B, ARS, GNS, LYPLA3, and GM2A, are all upregulated in protein expression (Figure 4A). The fold changes of these proteins in PC9/GR cells versus PC9 cells are listed in Table S5, Supporting Information.

The endocytic pathway is enriched with ubiquitylated proteins in resistant cells. For example, proteins involved in early endosome and late endosome formation, such as RAB5C, RAB7A, RAB11B, CHMP2A, MVB12B, VPS37A, TSG101, and in clathrin dependent and independent endocytosis include TGFBR1, PLD1, LDLR, NEDD4L, and CAV2. Specifically, Rab family GTPases regulate many steps of membrane traffic, in which cell surface proteins are trafficked from the Golgi to the plasma membrane and are recycled^19^ (Figure 4B). The fold change in ubiquitylation of these proteins at specific sites is listed in Table S5, Supporting Information.

The lysosomal pathway is also enriched with ubiquitylated proteins in gefitinib‐resistant cells, which include lysosomal membrane proteins including ATP6V0A4, ATP6AP1, CLN3, CTN7, LAPTM4A, ABCA2, MFSD8, and SLC17A5, and proteins involved in the transport of synthesized lysosomal enzymes among Golgi body, late endosome, and lysosome: AP1G1, CLTA, and CLTC. Among them, ATP6V0A4 is a component of vacuolar ATPase (V‐ATPase) that mediates acidification of intracellular compartments of eukaryotic cells important for protein sorting and endocytosis. CLTA is the major protein of the polyhedral coat of coated pits and vesicles during endocytosis (Figure 4C). The fold change in ubiquitylation of these proteins at specific sites is listed in Table S5, Supporting Information.

These findings indicate that both protein expression and ubiquitylation are upregulated in lysosomal and endocytic pathways during gefitinib resistance.

### 3.5 Ubiquitylation Sites in Proteins are Revealed by DiGly Proteomics

**Figure 5:**
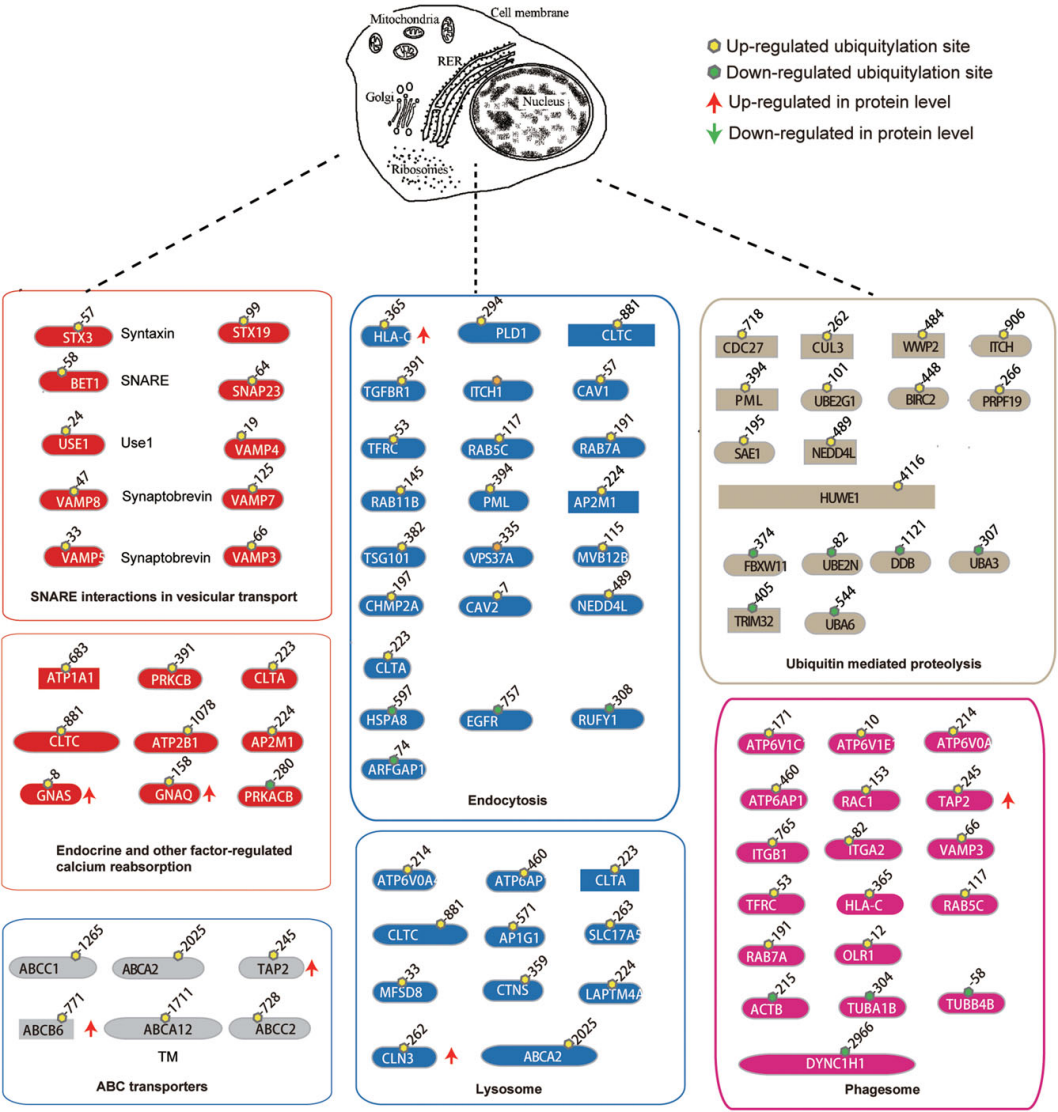
Ubiquitylated sites in proteins revealed by diGly proteomics in PC9/GR cells. Ubiquitylated proteins are listed in the following pathways: for SNARE interactions in vesicular transport pathway (red), the ubiquitylated targets are STX3, STX19, BET1, USE1, SNAP23, VAMP8, VAMP4, VAMP5, VAMP7, and VAMP3; for the endocytosis pathway (blue), the target proteins are: PLD1, HSPA8, EGFR, CLTA, CLTC, TGFBR1, ITCH, ITCH, CAV1, TFRC, HLA‐C, RAB5C, RAB7A,RAB11B, PML, AP2M1,TSG101, VPS37A, MVB12B, CHMP2A,LDLR, RUFY1, ARFGAP1, CAV2, and NEDD4L; for the lysosome pathway (blue), they are: ATP6V0A4, ATP6AP1, CLTA, CLTC, ABCA2, SLC17A5, MFSD8, CTNS, LAPTM4A, CLN3, and AP1G1; for the ABC transporter pathway (grey), they are: ABCA2, ABCA12, TAP2, ABCB6, ABCC1, ABCC2, and ABCC4; for the phagosome pathway (pink), the targets are: ATP6V1C1, ATP6V1E1, ATP6V0A4, ATP6AP1, RAC1, TAP2, ACTB, ITGB1, ITGA2, TFRC, HLA‐C, TUBA1B, TUBB4B, RAB5C, RAB7A, OLR1, DYNC1H1, and VAMP3; for the ubiquitin mediate proteolysis pathway (brown), the target proteins are: CDC27, FBXW11, CUL3, WWP2, ITCH, ITCH, PML, UBE2G1, UBE2N, HUWE1, PRPF19, TRIM32, DDB1, SAE1, UBA3, UBA6, NEDD4L, and BIRC2; for the endocrine and other factor‐regulated calcium reabsorption pathway (red), the targets are: ATP1A1, PRKCB, PRKACB, GNAS, GNAQ, CLTA, CLTC, ATP2B1, and AP2M1. Dotted lines indicated cell organelle where the pathways function. ↑and↓ indicate protein level decreases or increases in gefitinib‐resistant PC9/GR cells.

This study has allowed us to identify hundreds of differentially regulated ubiquitylated lysine sites of proteins in both PC9/GR and PC9 cells. For the top eight pathways significantly enriched in proteins with increased ubiquitylated lysine sites in gefitinib‐resistant cells, the specific lysine position within the protein, and the extent of ubiquitylation change (log2 ratio (L:H)) in PC9/GR cells compared to PC9 cells are listed in Table S6, Supporting Information. In addition, the schematic drawings of these proteins carrying ubiquitylated lysine sites within these pathways are also presented in Figure 5A.

We also found that many lysine sites in E3 ubiquitin ligases are differentially regulated by ubiquitylation in PC9/GR cells (Figure S5a, Supporting Information). Protein ubiquitylation in most of these enzymes is not associated with changes in the protein expression level, except DZIP3, TRAF3, and RNF149, RBBP4 proteins. Interestingly, TRIM32 and COPA have different lysine sites undergoing both up‐ and downregulation in ubiquitylation at the same time in PC9/GR cells. However, it is possible that some of the differentially expressed protein were not detected by the current LC‐MS/MS method.

Furthermore, many of autophagy related proteins have either differentially regulated protein ubiquitylation or protein expression (Figure S5b, Supporting Information). For example, NBR1 protein has the most significant increase in the ubiquitylation level, while HIF1A protein has the most significant decrease in the ubiquitylation level. LAMP2, GABARAPL2 have upregulated protein expression level, while ATG7, RB1CC1 have downregulated protein expression level.

Taken together, our study reveals hundreds of lysine sites in proteins that are differentially regulated by ubiquitylation in gefitinib‐resistant cells, which may regulate gefitinib resistance.

### 3.6 HMGA2 Overexpression or Alox5 Knockdown Suppresses Gefitinib Resistance by Repressing Autophagy

To validate whether differentially expressed proteins identified in our proteomic study are important in regulating gefitinib resistance, we chose HMGA2 and ALOX5 for subsequent validation, because HMGA2 is a known driver promoting tumor metastasis and a molecular target in many cancers,^20^ but it is significantly downregulated in gefitinib‐resistant PC9/GR cells (Figure 1C). While ALOX5 is also aberrantly expressed in several tumor types, it serves as a therapeutic target in acute myeloid leukemia,^21^ and it is upregulated in gefitinib‐resistant PC9/GR cells. Therefore, understanding how these genes function in gefitinib resistance is important for developing effective treatment for lung cancer. We generated PC9/GR cell lines stably overexpressing the control plasmid, HMGA2, or ALOX5 cDNA, or cells stably expressing the control shRNA, or shRNAs targeting HMGA2 or ALOX5.

**Figure 6:**
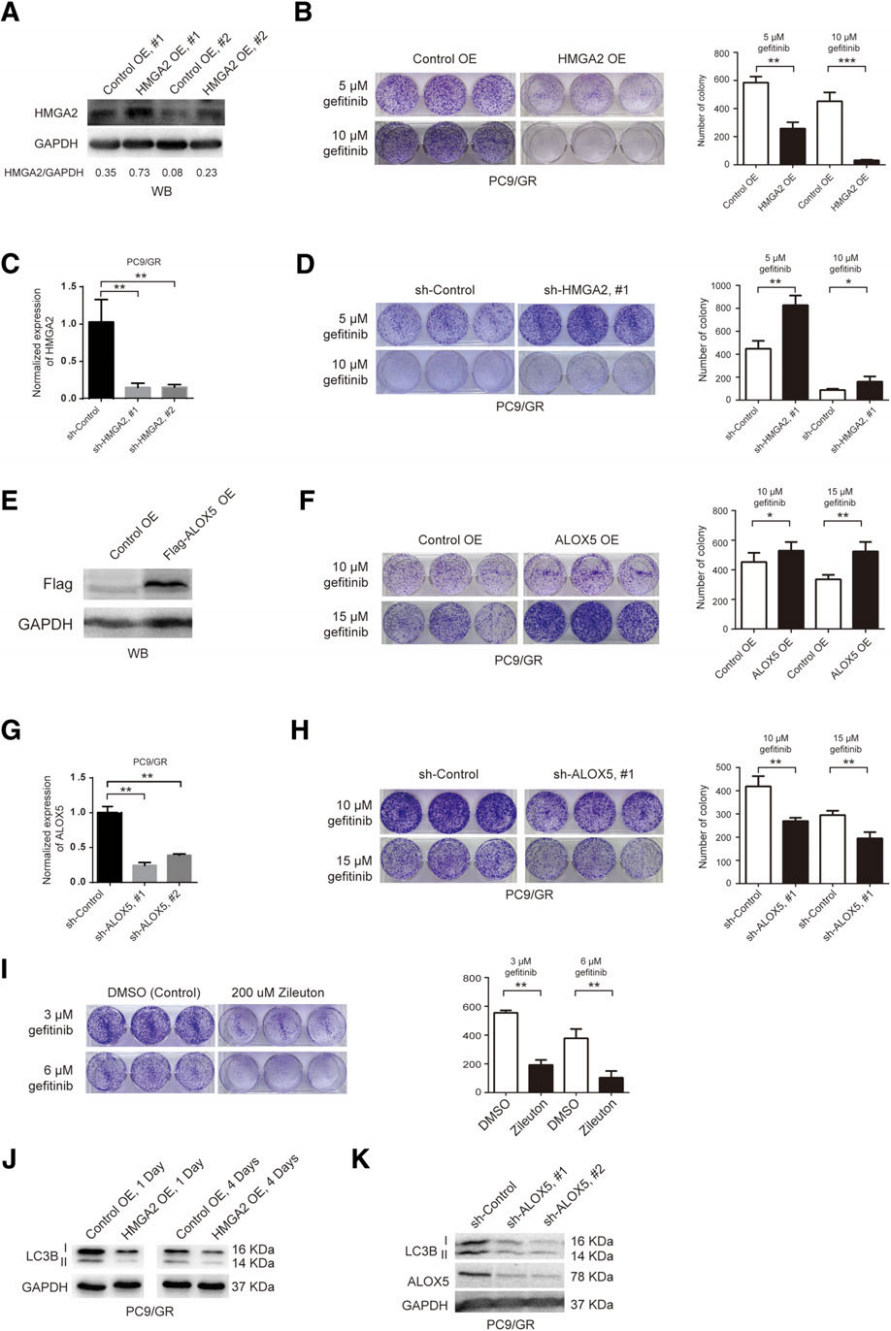
Overexpression of HMAG2 or knocking down of ALOX5 suppresses gefitinib resistance by inhibiting autophagy. A) WB validation of HMAG2 overexpression in PC9/GR cells stably expressing the control (Control OE, #1 and #2) or HMGA2 overexpression (HMGA2 OE, #1 and #2) plasmids. The ratio of band intensity of HMGA2 versus GAPDH (HMGA2/GAPDH) was shown underneath each condition. B) Comparison of gefitinib‐resistant colony formation between PC9/GR cells stably expressing the control or HMGA2 overexpression plasmid. PC9/GR cells were cultured in 5 or 10 μm gefitinib. The number of colonies is quantified in the bar graph on the right side. Each condition was repeated in triplicates. This is true for all the rest of the experiments. C) Quantification of HMAG2 knockdown level in PC9/GR cells stably expressing the control (sh‐Control) or shRNA targeting HMGA2 (sh‐HMGA2, #1 and #2) by RT‐qPCR. D) Comparison of gefitinib‐resistant colony formation between PC9/GR cells stably expressing sh‐Control or sh‐HMGA2 #1, cultured in 5 or 10 μm gefitinib. E) WB validation of flag tagged ALOX5 overexpression in PC9/GR cells stably expressing the control (Control OE) or ALOX5 overexpression (Flag‐ALOX5 OE) plasmid. F) Comparison of gefitinib‐resistant colony formation between PC9/GR cells stably expressing the control or ALOX5 overexpression plasmid. G) Quantification of ALOX5 knockdown level in PC9/GR cells stably expressing the control (sh‐Control) or shRNA targeting ALOX5 (sh‐ ALOX5, #1 and #2) by RT‐qPCR. H) Comparison of gefitinib‐resistant colony formation between PC9/GR cells stably expressing sh‐Control or sh‐ ALOX5 #1. I) Comparison of gefitinib‐resistant colony formation between PC9/GR cells treated with the control (DMSO) or 200 um Zileuton. J) WB detection of LC3B II (autophagy marker) expression in PC9/GR cells overexpressing control (Control OE) or HMGA2 (HMGA2 OE) plasmid at 1 or 4 days. K) WB detection of LC3B and ALOX5 expression in PC9/GR cells overexpressing the control shRNA (sh‐Control) or shRNA targeting ALOX5 (sh‐ALOX5, #1 and #2).

Cells stably overexpressing HMGA2 cDNA showed the higher expression of HMGA2 protein compared to the control by WB (Figure 6A), and reduced gefitinib resistance at 5 or 10 μm gefitinib concentration (Figure 6B). Conversely, when PC9/GR cells stably express shRNA against HMGA2, HMGA2 expression was reduced greatly compared to the control shRNA (Figure 6C), gefitinib resistance was enhanced in these cells (Figure 6D), indicating HMGA2 upregulation inhibits gefitinib resistance, while its downregulation promotes gefitinib resistance.

To study whether ALOX5 is involved in gefitinib resistance, we overexpressed the flag tagged ALOX5 cDNA in PC9/GR cells, and verified its overexpression by WB (Figure 6E), and these cells exhibited enhanced gefitinib resistance, compared to the control, at 10 or 15 μm gefitinib concentration (Figure 6F). Conversely, when ALOX5 was stably knocked down in PC9/GR cells, its expression was significantly reduced, which was validated by RT‐qPCR (Figure 6G), the number of gefitinib‐resistant colonies also decreased dramatically, compared to the control (Figure 6H). Furthermore, under the treatment of 200 μm zileuton, a well‐known ALOX5 small molecule inhibitor,^22^ the number of gefitinib‐resistant colonies was also greatly reduced (Figure 6I), indicating ALOX5 overexpression promotes gefitinib resistance, while ALOX5 knockdown or inactivation suppresses gefitinib resistance.

In our previous study, we found that autophagy is significantly activated in gefitinib‐resistant cells, and inhibition of autophagy reduces gefitinib resistance.^23^ To study whether HMGA2 overexpression or ALOX5 knockdown affects autophagy, we carried out WB assays to compare the expression level of LC3B II, the active form of LC3B protein, which can be used as an indicator of autophagy, among different conditions, and found that LC3B II expression was decreased upon HMGA2 overexpression (Figure 6A,J) or ALOX5 knockdown (Figure 6K) in PC9/GR cells, compared to the control, indicating that HMGA2 overexpression or ALOX5 knockdown may suppress gefitinib resistance by inhibiting autophagy.

**Figure 7:**
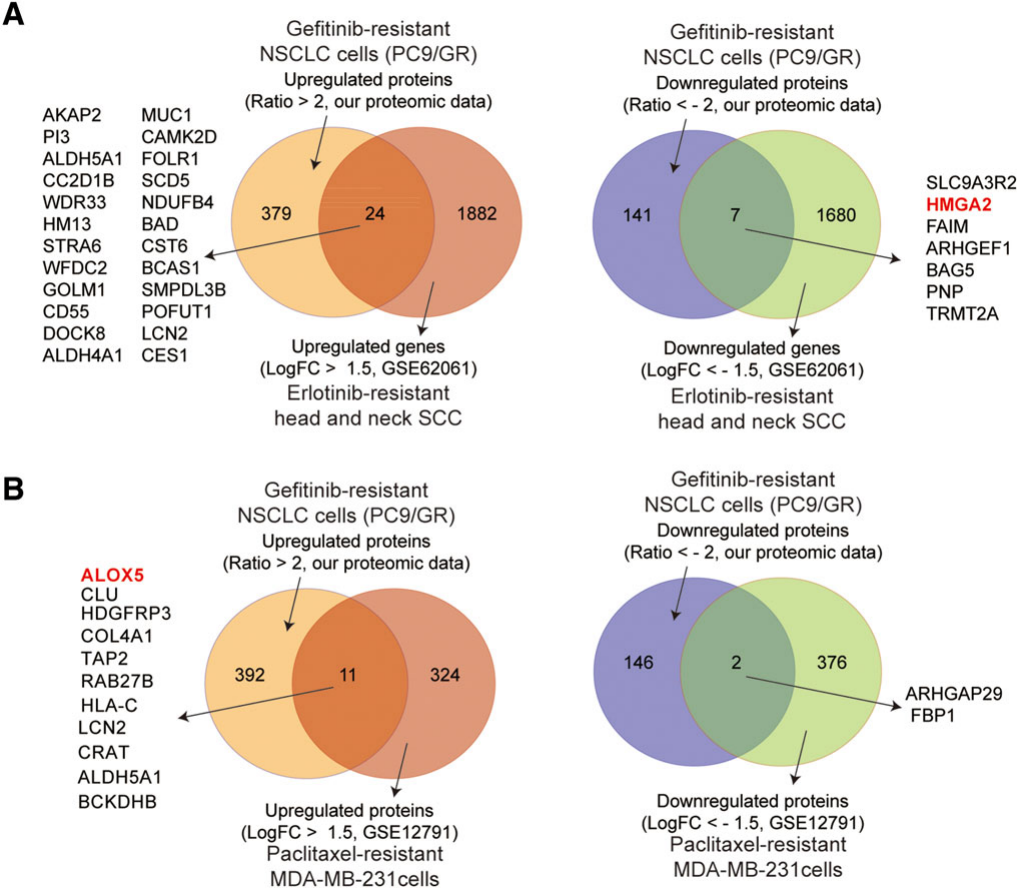
Proteins or genes found up‐ or downregulated in drug‐resistant cancer cells from other studies. A) Proteins or genes found upregulated (left panel) or downregulated (right panel) in PC9/GR cells (from our proteomic study) and erlotinib‐resistant head and neck SCC cells (from the GSE62061 study) by Venn diagram analysis. B) Proteins or genes found upregulated (left panel) or downregulated (right panel) in PC9/GR cells (from our proteomic study) and paclitaxel‐resistant MDA‐MB‐231 cells (from the GSE12791 study) by Venn diagram analysis. The proteins or genes shared by both studies were listed besides the Venn diagram. HMGA2 and ALOX5 are highlighted in red.

Finally, to find out whether HMGA2 downregulation or ALOX5 upregulation can also be observed in other types of drug‐resistant cells, we searched the GEO database (https://www.ncbi.nlm.nih.gov/geo/), and found that HMGA2 is downregulated in erlotinib‐resistant head and neck squamous cell carcinoma cells in GSE62061 dataset (Figure 7A), while ALOX5 upregulation is found in paclitaxel‐resistant MDA‐MB‐231 breast cancer cells (Figure 7B), indicating there may be common mechanisms of drug resistance in these cases.

## 4 Discussion

In the current study, we have quantified changes in the expression level of 3773 proteins and changes in the ubiquitylation level of 2893 lysine sites in 1415 proteins in both gefitinib‐resistant and sensitive NSCLC cells, revealed distinctive cellular pathways associated with these changes in resistant cells. Specially, lysosomal and endocytic pathways are enriched with either a large number of upregulated proteins or protein ubiquitylation. In addition, HMGA2 overexpression or ALOX5 inactivation suppresses gefitinib resistance by inhibiting autophagy.

There are several technical improvements presented in this work, compared to previous published data.^24^ First, this is the first study of changes in global ubiquitylome associated with gefitinib resistance; second, we used SILAC technology to quantify global changes in protein expression and ubiquitylation, which is more accurate and has a higher throughput than other technologies, such as, 2D differential gel electrophoresis (2D‐DIGE), followed by matrix‐assisted laser desorption ionization time‐of‐flight mass spectrometry (MALDI‐TOF MS) analysis.^24^

In addition, there are several novel findings coming out of our study. First, it reveals that both protein expression and ubiquitylation are upregulated in lysosomal and endocytic pathways during gefitinib resistance, implying that ubiquitylation of these proteins may be necessary for the proper function or localization of these proteins, other than the degradation of these proteins. This is consistent with previous studies showing that a majority of the diGly‐containing proteome does not represent conventional proteasome substrates, indicating ubiquitylation exerts functions other than degradation of target proteins.^25^

Furthermore, we found that, in gefitinib‐resistant cells, ubiquitylated proteins are significantly enriched in pathways, including SNARE interactions in vesicular transport, phagosome, ABC transporters, and cell adhesion molecule (CAMs pathways. Even though these pathways are previously implicated in drug resistance, for example, ABC transporters are upregulated during drug resistance.^26^ Autophagy is enhanced during the drug resistance,^27^ endocytosis, phagosome and CAMs are also implicated in drug resistance,^28^ but the enhanced ubiquitylation of these proteins associated with gefitinib resistance was not reported before.

Finally, we have shown that, HMGA2 expression was decreased dramatically in gefitinib‐resistant cells, as well as in erlotinib‐resistant head and neck squamous cell carcinoma cells. In contrast, ALOX5 expression was increased significantly in gefitinib‐resistant cells, as well as paclitaxel‐resistant breast adenocarcinoma cells (MDA‐MB‐231). In addition, HMGA2 overexpression or ALOX5 knockdown suppresses gefitinib resistance possibly by inhibiting autophagy. Therefore, these results not only validate our proteomics findings, but also indicate that ALOX5 may serve as a potential therapeutic target for overcoming gefitinib resistance. More detailed study of how ALOX5 inhibition suppresses gefitinib resistance is currently under investigation.

Overall, our study reveals the previously unknown global landscape of changes in protein expression and ubiquitylation, especially in lysosomal and endocytic pathways, during gefitinib resistance, and further demonstrates that HMGA2 overexpression or ALOX5 knockdown can suppress gefitinib resistance possibly by inhibiting autophagy. Therefore, this study may help to identify new therapeutic targets to overcome gefitinib resistance in NSCLC.

## Conflict of Interest

The authors declare no conflict of interest.

## Supporting information

Supporting Information

Supporting Information

Supporting Information

Supporting Information

Supporting Information

Supporting Information

Supporting Information

Supporting Information

Supporting Information

Supporting Information

Supporting Information
