# Supplementary figures and images for: Integrative Analysis of Proteome and Ubiquitylome Reveals Unique Features of Lysosomal and Endocytic Pathways in Gefitinib‐Resistant Non‐Small Cell Lung Cancer Cells
Source: Proteomics. 2018 Jul 8;18(15):1700388. doi: 10.1002/pmic.201700388 (PMC6099292; doi:10.1002/pmic.201700388)

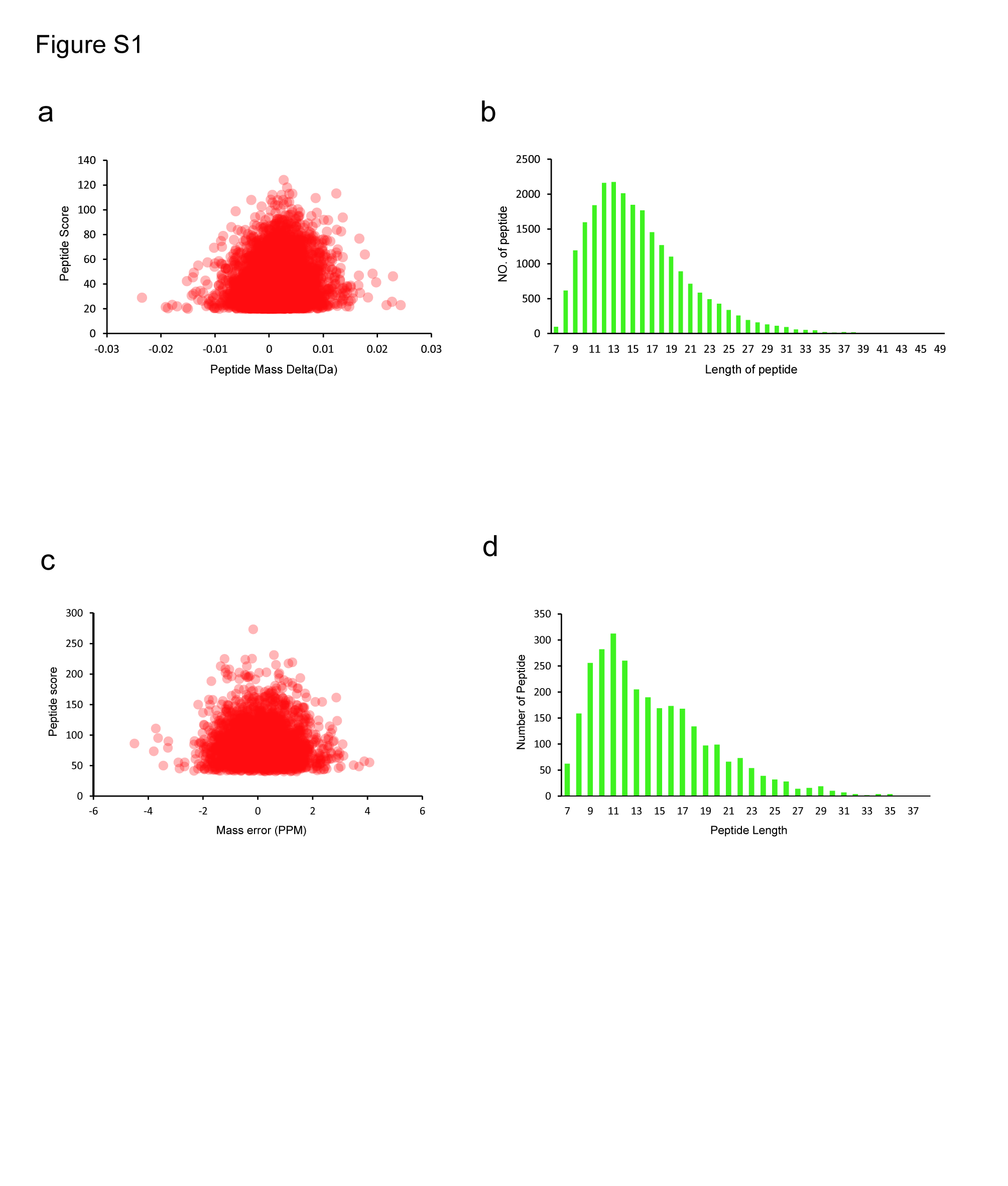

Supplement: Supplementary file 1 — Supporting Information [file PMIC-18-na-s001.tif]

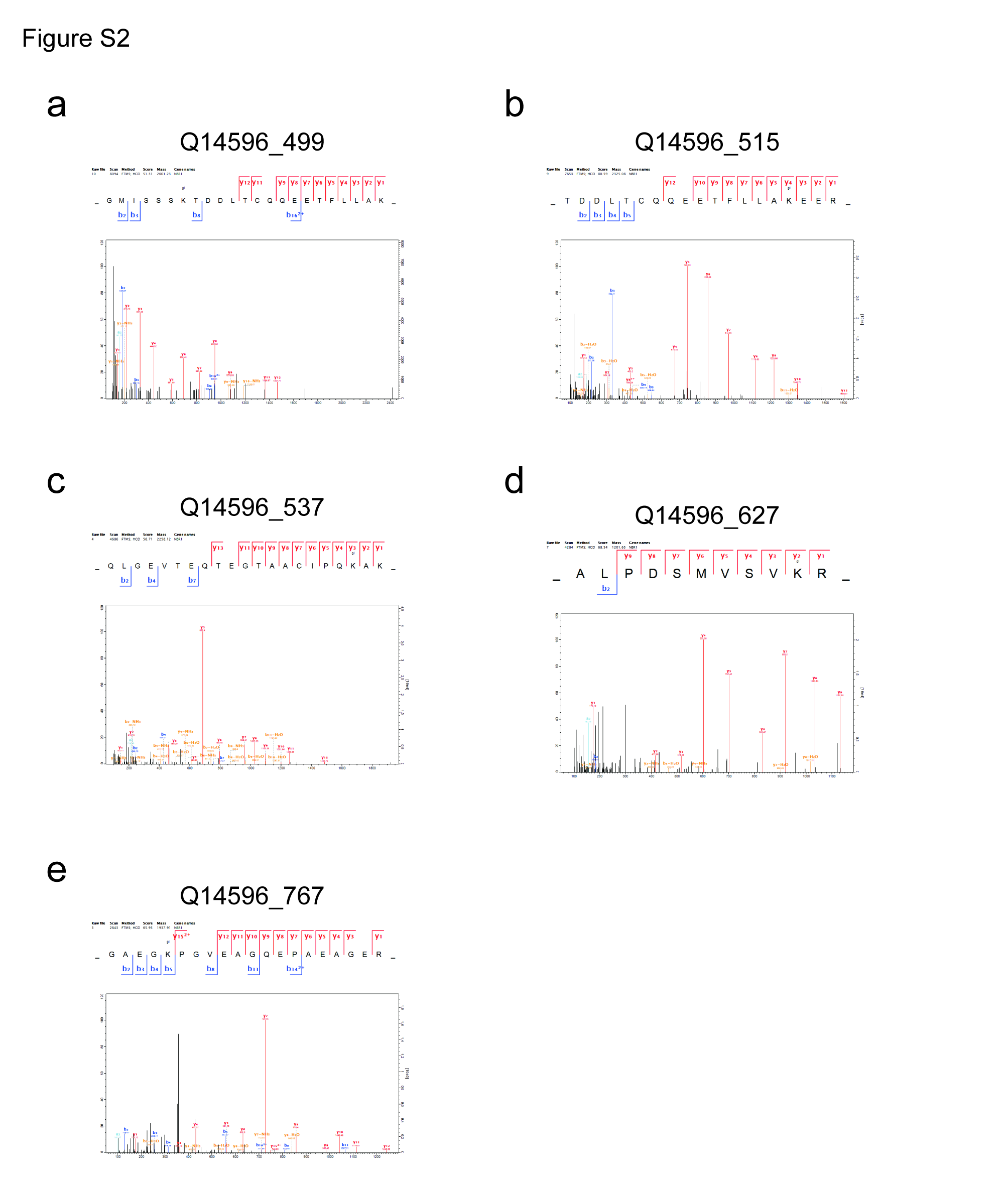

Supplement: Supplementary file 2 — Supporting Information [file PMIC-18-na-s002.tif]

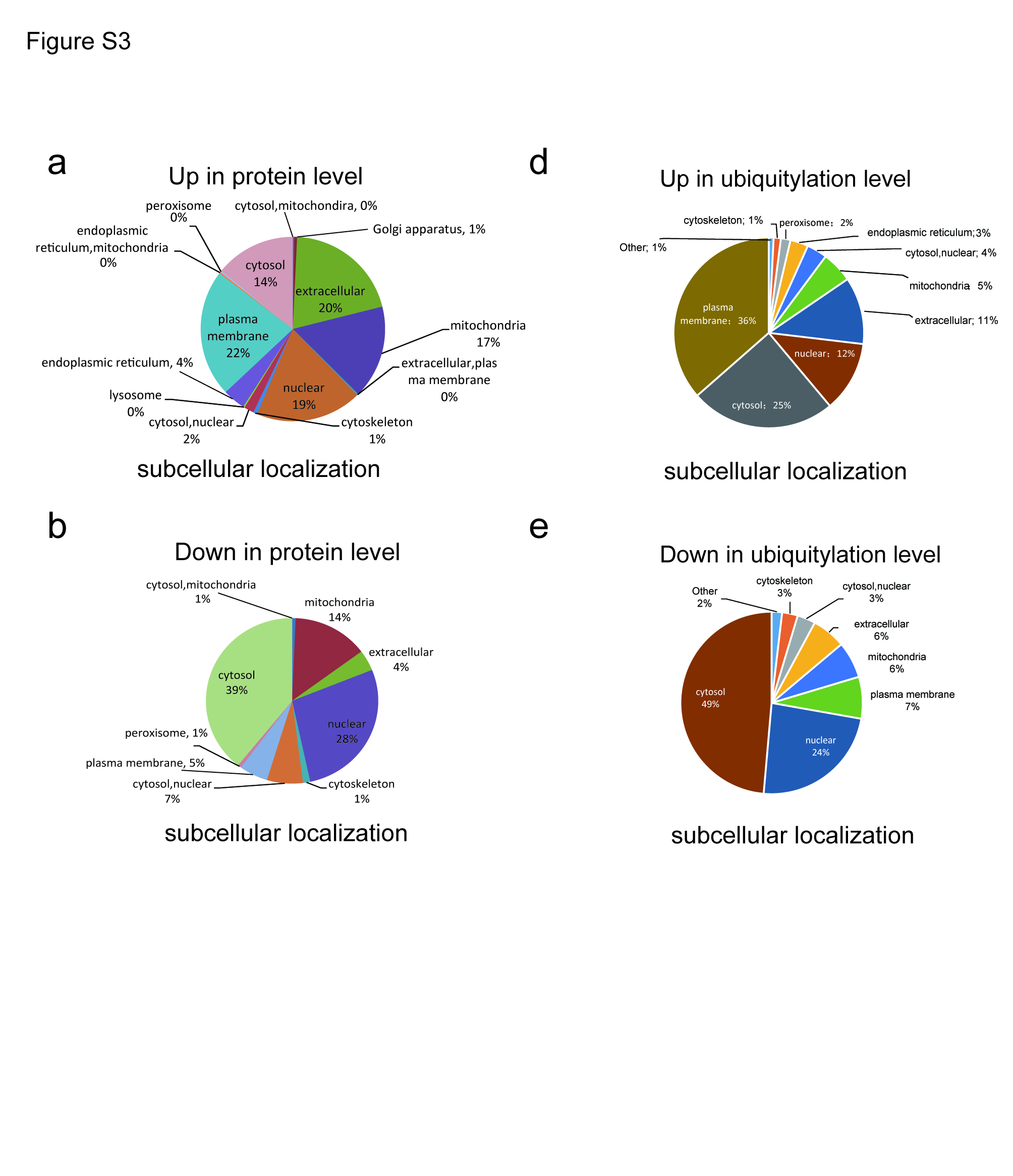

Supplement: Supplementary file 3 — Supporting Information [file PMIC-18-na-s003.tif]

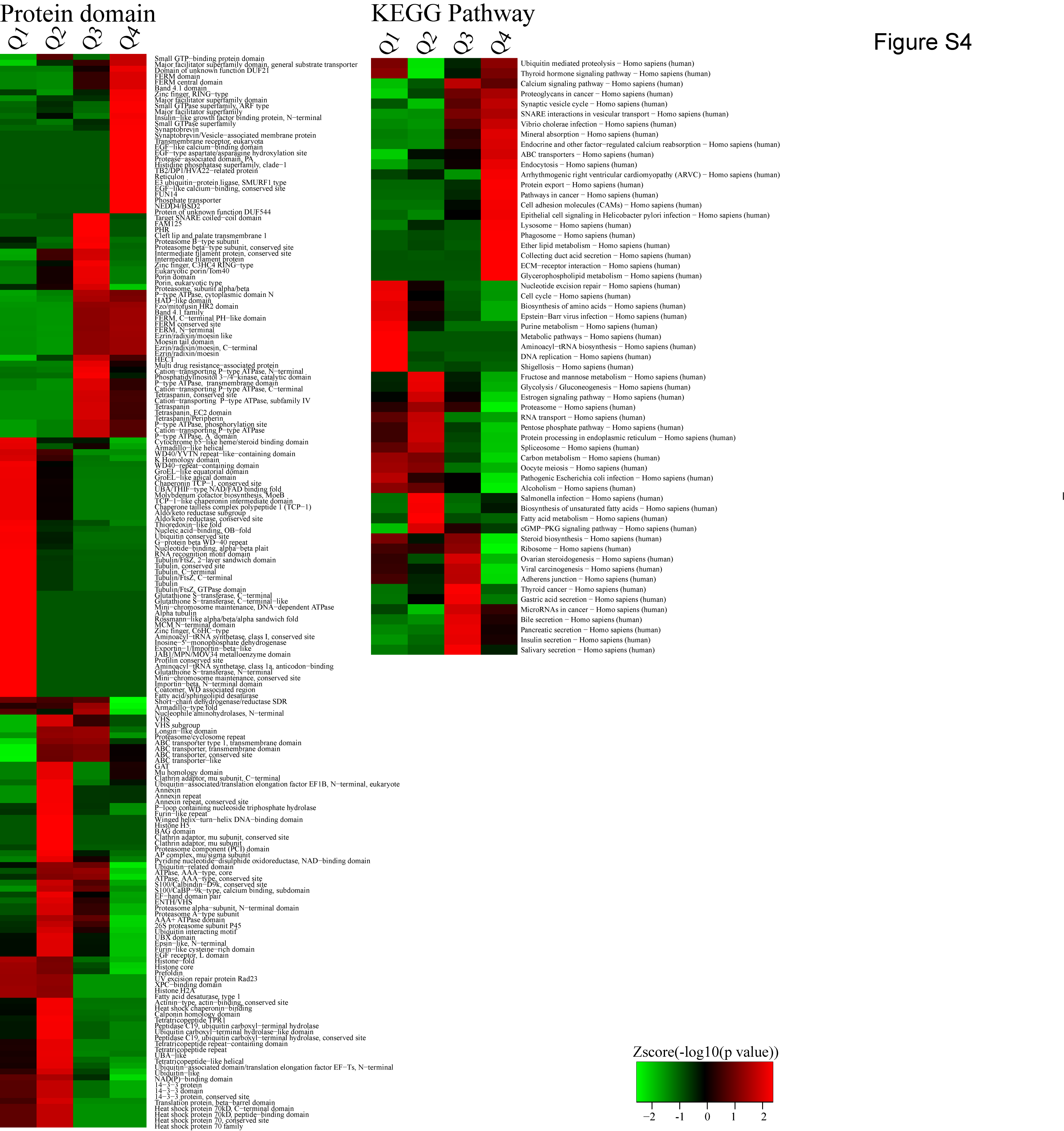

Supplement: Supplementary file 4 — Supporting Information [file PMIC-18-na-s004.tif]

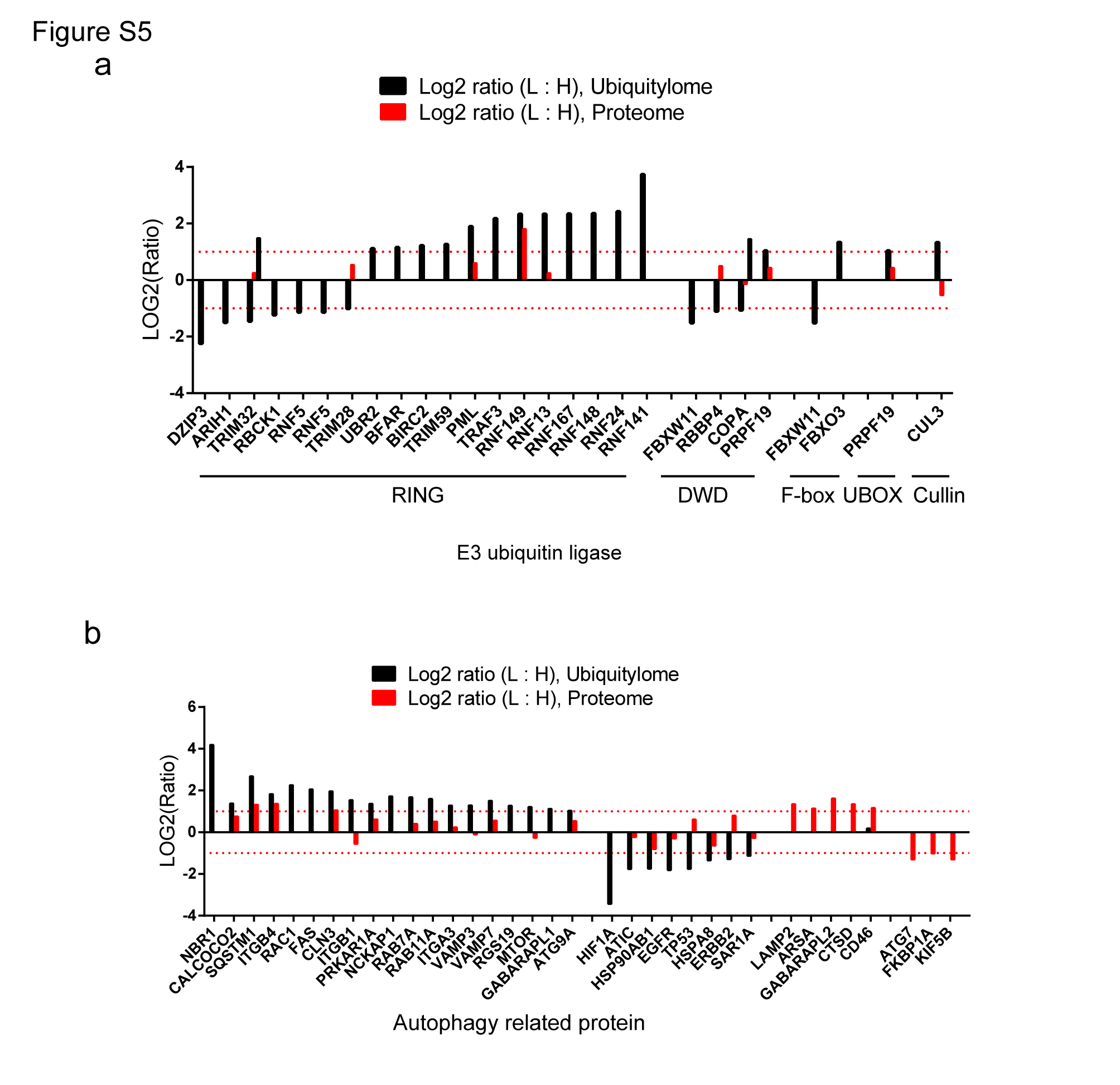

Supplement: Supplementary file 5 — Supporting Information [file PMIC-18-na-s005.tif]
