# Supplementary material for: Integrative Analysis of Proteome and Ubiquitylome Reveals Unique Features of Lysosomal and Endocytic Pathways in Gefitinib‐Resistant Non‐Small Cell Lung Cancer Cells
Source: Proteomics. 2018 Jul 8;18(15):1700388. doi: 10.1002/pmic.201700388 (PMC6099292; doi:10.1002/pmic.201700388)
Supplement: Supplementary file 6 — Supporting Information [file PMIC-18-na-s006.docx]

**Integrative analysis of proteome and ubiquitylome reveals unique features of lysosomal and endocytic pathways in gefitinib resistant non-small cell lung cancer cells**

**Supplementary figure legends:**

**Figure S1. QC Validation of the MS data in the proteome and ubiquitylome studies.** (a)

For the proteome study, the distribution of mass error is near zero and most of them are less than 0.02 Da which means the mass accuracy of the MS data fit the requirement. (b) For the proteome study, the length of most peptides is distributed between 8 and 16, which agrees with the property of tryptic peptides, and means that sample preparation reaches the standard. (c) For the ubiquitylome study, the distribution of mass error of all the identified peptides is near zero and most of them are less than 2 PPM which means the mass accuracy of the MS data fit the requirement. (d) For the ubiquitylome study, the distribution of the length of most peptides is between 8 and 20, which agrees with the property of tryptic peptides, and means that sample preparation reaches the standard.

**Figure S2. Representative MS spectra using peptides identified in NBR1 protein (Q14596) in PC9/GR cells as an example.** (a, b, c, d, e) For NBR1 protein, there are five identified peptides containing lysine residues that were ubiquitylated at the following positions: 499, 515, 537, 627, and 767, in PC9/GR cells. The x axis represents the ratio of M/Z, the left y axis represents the relative intensity (%) of peaks, and the right y axis represents intensity (10e4). Peptides broken from the left were labeled as blue peaks, and those broken from the right were labeled as red peaks. Those broken peptides forming the complex with water were labeled as yellow peaks, and other broken peptides unable to be identified were labeled as black peaks.

**Figure S3. Classification of subcellular localization for up- or down-regulated proteins or protein ubiquitylation in PC9/GR vs. PC9 cells.** (a, b) Subcellular localization for up- (a) or down- (b) regulated proteins in PC9/GR cells. (c, d) Subcellular localization for proteins with lysine sites undergoing up- (c) or down (d) –regulated ubiquitylation in PC9/GR cells.

**Figure S4. Heatmap of protein domain or KEGG pathway clustering for protein groups undergoing changes in ubiquitylation in PC9/GR vs. PC9 cells.** (a, b) Protein domain (a) or KEGG pathway clustering (b) for protein groups (Q1, Q2, Q3, and Q4) identified in the ubiquitylome study, which are described in the figure 1b. Z score = -log10 (p value). Green represents the negative z score, and red represents the positive z score.

**Figure S5. Comparison of changes in protein expression and ubiquitylation in E3 ubiquitin ligases and autophagy related proteins in PC9/GR vs. PC9 cells.** (a) E3 ubiquitn ligases, which undergo up- or down-regulation in protein ubiquitylation in PC9/GR cells (Labeled as “L”) vs. PC9 cells (Labeled as “H”), are divided into four groups according to the functional domain: RING, DWD, F-box, UBOX, and Cullin. Black bar represents log2 ratio (L : H) from the ubiquitylome study, red bar represents log2 ratio (L : H) from the proteome study. Dashed red line represents log2 ratio at + 1 or – 1. (b) Autophagy related proteins are divided into four groups: upregulated (group 1) or downregulated (group 2) in protein ubiquitylation, and upregulated (group 3) or downregulated (group 4) in protein expression, in PC9/GR cells vs. PC9 cells. All bars and lines represent the same meanings as in (a). Note: ITGB4 have eight lysine sites that are differentially ubiquitylated in PC9/GR cells, but only one of these sites is shown here.

**Supplementary tables:**

**Table S1. Peptide identification in both PC9/GR cells and PC9 cells in the proteome study.**

PC9/GR cells were labeled with “light isotopic lysine” (12C-Lysine) or “L” in the table, and PC9 cells were labeled with “heavy isotopic lysine” (13C-Lysine) or “H” in the table using a SILAC protein quantitation kit.

**Table S2. Identified and quantified proteins in both PC9/GR cells and PC9 cells in the proteome study.**

**Table S3. Identified and quantified proteins with lysine sites undergoing changes in ubiquitylation in both PC9/GR cells and PC9 cells in the ubiquitylome study.**

**Table S4. Pathway enrichment analysis for quantified proteins and ubiquitylated proteins in both PC9/GR cells and PC9 cells.**

**Table S5. Fold changes in protein expression or ubiquitylation in both PC9/GR cells and PC9 cells.**

**Table S6. Identification of lysine sites undergoing up-regulation in ubiquitylation in specific pathways in PC9/GR cells.**

**Supplementary methods:**

**Pathway analysis in the proteome study**

The database for annotation, visualization and integrated discovery (DAVID) v6.7 was used to find out the significant biological pathways (KEGG pathways) associated with the differentially expressed genes in both the control and experiment groups [[1](#_ENREF_1), [2](#_ENREF_2)].

**Cell culture**

Two human NSCLC cells, PC9 and PC9/GR, were grown in DMEM medium (Hyclone, GE Healthcare Life Sciences, Utah, USA). All medium contained 10% FBS (Gibco) supplemented with penicillin (100 U/ml) and streptomycin (100 mg/ml) (Life Technologies). Gefitinib was added to the medium of PC9/GR cells at a final concentration of 3 µM. Cells were incubated at 37°C in a humidified atmosphere with 5% CO2.

**Reference:**

[1] Huang da, W., Sherman, B. T., Lempicki, R. A., Systematic and integrative analysis of large gene lists using DAVID bioinformatics resources. *Nature protocols* 2009, *4*, 44-57.

[2] Huang da, W., Sherman, B. T., Lempicki, R. A., Bioinformatics enrichment tools: paths toward the comprehensive functional analysis of large gene lists. *Nucleic acids research* 2009, *37*, 1-13.
